# Initial experiences of prehospital blood product transfusions between 2016 and 2020 in Päijät-Häme hospital district, Finland

**DOI:** 10.1186/s13049-022-01027-z

**Published:** 2022-06-06

**Authors:** Heidi Yliharju, Timo Jama, Hilla Nordquist

**Affiliations:** 1 Emergency Medical Service, Päijät-Häme Joint Authority for Health and Wellbeing, Keskussairaalankatu 7, 15850 Lahti, Finland; 2 Department of Health care and Emergency care, South-Eastern Finland University of Applied Sciences, Pääskysentie 1, 48220 Kotka, Finland

**Keywords:** Prehospital blood product transfusions, Freeze-dried plasma, Packed red blood cells, Remote damage control resuscitation, Non-trauma, Trauma, Ground-based emergency medical services

## Abstract

**Background:**

Treating hemorrhaging patients with prehospital blood product transfusions (PHBT) narrows transfusion delays and potentially benefits the patient. We describe our initial experiences of PHBT in a ground-based emergency medical service (EMS), where the transfusion protocol covers both traumatic and nontraumatic hemorrhaging patients.

**Methods:**

A descriptive retrospective analysis was performed on the records of all the patients receiving red blood cells, freeze-dried plasma, or both during prehospital care from September 2016 to December 2020. The delays of PHBT and the effects on patients’ vital signs were analyzed and reported as the median and interquartile range (IQR) and analyzed using a Wilcoxon Signed rank test.

**Results:**

65 patients received prehospital blood product transfusions (PHBT), 29 (45%) were non-traumatic, and 36 (55%) traumatic. The main two reasons for PHBT were blunt trauma (n = 30, 46%) and gastrointestinal hemorrhage (n = 20, 31%). The median time from the emergency call to the start of PHBT was 54 min (IQR 38), and the transfusion began on a median of 61 min (IQR 42) before arrival at the hospital. The median systolic blood pressure improved from a median 76.5 mmHg (IQR 36.5) before transfusion to a median of 116.60 mmHg (IQR 26.5) (p < 0.001) on arrival to the hospital. No transfusion-related severe adverse events were noted.

**Conclusions:**

Starting PHBT in ground-based EMS is a feasible and viable option. The PHBT began significantly earlier than it would have started on arrival to the hospital, and it seems to be safe and improve patients’ physiology.

**Study approval:**

D/2603/07.01.04.05/2019.

## Background

Treating hemorrhaging trauma patients has shifted from crystalloid resuscitation towards balanced prehospital blood product transfusions (PHBT) [1]. Using blood components in the prehospital setting can shorten the timeframe for transfusions significantly, [2–4] and patients arrive at the hospital with improved hemodynamic parameters [5]. For severely injured patients, 1:1-ratio prehospital transfusion of plasma and packed red blood cells (pRBCs) seems to improve their chances of survival [5, 6] and earlier transfusions can decrease the need for additional transfusions during the first 24 h of care [5, 7–9].

In addition, non-traumatic hemorrhaging patients can also form a significant group that could benefit from prehospital transfusions [10–12]. Causes for non-traumatic hemorrhaging can include different reasons for postoperative complications, gastrointestinal hemorrhaging, and obstetric and gynecological emergencies. The transfusion criteria are usually the same for both traumatic and non-traumatic patients. However, according to patient, the trauma patients are typically younger and have profound shock, while the non-traumatic patients are typically older and more anemic. [10, 12] Earlier PHBT seem to correct anemia and coagulopathy in non-traumatic patients [11].

In the Finnish hospital district area of Päijät-Häme, the current prehospital transfusion protocol has been in use since fall 2016, and it covers both traumatic and non-traumatic hemorrhaging patients. The prior research has mainly focused on presenting PHBT on trauma patients treated by Helicopter Emergency Medical Services (HEMS) and research presenting ground-based EMS or non-traumatic and/or mixed patient groups is limited. Thus, we primarily aimed to describe the patients that received PHBT in ground-based EMS with long transport times and secondary we aimed to analyze PHBTs’ effect for patients’ physiology and further transfusion need. We analyzed the patient characteristics, both traumatic and non-traumatic, and their response to the prehospital blood product transfusions, and the timeframe prehospital transfusion began.

## Methods

### Setting

A descriptive retrospective analysis was performed on the patient records of all the patients receiving PHBT (pRBCs, freeze-dried plasma (FDP, LyoPlas AB, Deutsches Rotes Kreutz Blutspendedienst, Germany), or both) in Päijät-Häme EMS, Finland, from September 1, 2016, to December 31, 2020 (= 52 months).

The Päijät-Häme central hospital district administers prehospital EMS for 230,000 people living in the Päijät-Häme region in Southern Finland. The Päijät-Häme Central Hospital is the second-largest central hospital in Finland and most patients in need of acute care (including intensive care) can be treated locally, but some of the critical patient care (large burns or massive head injuries) is centralized into the five university hospitals. Patients suspected of needing more specialized care can be transported from the scene straight to the university hospital.

The region’s prehospital EMS responses approximately 42,000 emergency calls, 3,500 hospital transports, and 1,500 other medical support tasks yearly. A doctor-based rapid response unit, staffed with an advanced level paramedic (with at least Bachelor’s degree in prehospital nursing) and a prehospital emergency care (PHEC) physician, is on call every day from 8 am to 8 pm. The dispatch of the rapid response PHEC physician unit is based on predefined criteria or upon request of an ambulance crew. During night hours (from 8 pm to 8 am), prehospital blood product transfusions are initiated by an EMS field supervisor after consultation with a university hospital-based HEMS physician.

### Prehospital transfusion protocol

The PHBT unit, carried by the rapid response PHEC physician unit during the day and EMS field supervisor unit during the night, consist of two units of type O RhD negative packed red blood cells and four units of freeze-dried plasma (FDP). The FDP in use is LyoPlas AB, Deutsches Rotes Kreutz Blutspendedienst, Germany. LyoPlas AB is made with AB-donors plasma and is compatible for all patients. The FDP is reconstructed with 200 ml of Aqua and equals 0.70–0.85 ml/ml of human plasma. Additionally, to the PHBT unit, three critical care ambulances in the area carry two units of freeze-dried plasma each and can start the blood product transfusion process before arrival of the red blood cells.

The guidelines for starting transfusion are similar in both trauma and non-traumatic patients: active major hemorrhage or suspicion of major hemorrhage with systolic blood pressure < 90 mmHg, or absent arterial radial pulse, or patient has symptoms of shock or when physician views that it would be beneficial for the patient. On arrival at the scene ambulance crew can suggest the PHBT for the rapid response unit physician or HEMS physician based on their clinical judgment.

In the PHBT protocol patient is transfused 1:1 with pRBCs and FDP. Before blood products patient is first given 1 g of tranexamic acid (TXA), second transfused with pRBCs and FDP and added to the protocol in the year 2020 calcium gluconate 10 mmol. The pRBCs are transfused through fluid warmer (MEQU M Warmer, MEQU Denmark) and the FDP is hung to gravity. Transfused amounts can variate, but for adults 1–2 units of pRBCs and 2–4 units of FDP are transfused. FDP can be transfused in suspected hemorrhage without pRBC transfusions with PHEC physicians’ decision.

The pRBCs can be changed or replenished any time of the day from the Päijät-Häme central hospital blood bank. The pRBCs are transported and stored in a temperature-controlled insulated box (Credo Promed, Pelican BioThermal, USA). The box has Seemoto censors for continuous temperature controlling (+ 2 °C to + 6 °C). Once a week pRBCs are replaced with new fresh units, and the previous units return to the blood bank, for circulation. All pRBC units are documented and tracked by the blood bank either transfused, waste, or back in stock. The rapid response unit crew prepares blood units for the transfusion and the ambulance crew prepares the patient for transfusion following their own checklists. All the area’s ambulances are equipped with a kit for blood drawing for ABO compatibility testing and the samples can be taken while inserting an intravenous line or it can be done separately.

### Study subjects and data collection

All the patients that had PHBT during the study period (September 2016–December 2020) were identified and included in the study. Three pediatric patients (< 13 years) were identified among the study subjects and are also included in the analysis. The data used in this study was collected from Päijät-Häme region electronic prehospital database (Codea Leda), Päijät-Häme Central Hospital blood bank records, and the Päijät-Häme Central Hospital electronic patient records (LifeCare). All patients receiving blood components in a prehospital setting were identified and included. For 6 patients (9%), there were no recognizable timestamps on when the prehospital transfusion started and for 10 patients the blood pressure was not measurable before transfusion, or the time stamps did not correlate with the blood transfusion and were excluded from the hemodynamic variables analysis but were included in other parts.

The following data was retrieved form the electronic prehospital database: dispatch and transportation codes and timestamps for emergency call, arrival of the first ambulance crew to the scene, start of PHBT and arrival to the hospital, patient characteristics (age, gender), reason for hemorrhage (non-traumatic/traumatic), prehospital vital signs before transfusion, prehospital blood transfusion protocol (pRBC, FDP, TXA, Calcium gluconate) and markings of transfusion safety and transfusion related adverse reactions (any mentions of transfusion-related acute lung injury, transfusion-associated circulatory overload, allergic reactions, acute hemolysis or febrile non-hemolytic transfusion reactions). The hospital blood bank records were evaluated and all the used and wasted pRBC units were counted. The hospital’s electronic patient record was reviewed, and the following data was retrieved: patient vital signs on arrival, first blood laboratory results (pH, BE, Lactate, Ca-ion, Hb), in-hospital blood transfusions during the first 6 h and 24 h and outcomes (mortality during hospital care, transfer for other care facility or discharge to home).

### Statistical methods

All data was collected in a Microsoft Office Excel Spreadsheet (Microsoft Corporation) and data analysis was performed on SPSS (IBM, version 27) for Windows. The analysis started by describing the study population. Descriptive statistics include frequencies and percentages, and were counted for all categorical variables (gender, age, trauma/non-trauma groups, PHBT). The effects of PHBT on patient vital signs were analyzed. D'Agostino-Pearson Test was used to test the normal distribution of the continuous variables. Because most of the variables had skewed distribution, continuous variables were reported as median, and interquartile range (IQR) and further analyzed using Wilcoxon Signed rank test. A p-value less than 0.05 was considered significant. Delays of PHBT were analyzed and noted as median and interquartile range. PHBT safety was analyzed by noting any adverse events in prehospital and hospital records and analyzing possible fever reactions on patient temperature changes before and after PHBT. In hospital blood transfusions and outcomes of the hospital stay were analyzed and reported as frequencies and percentiles.

## Results

### Patient characteristics

**Fig. 1:**
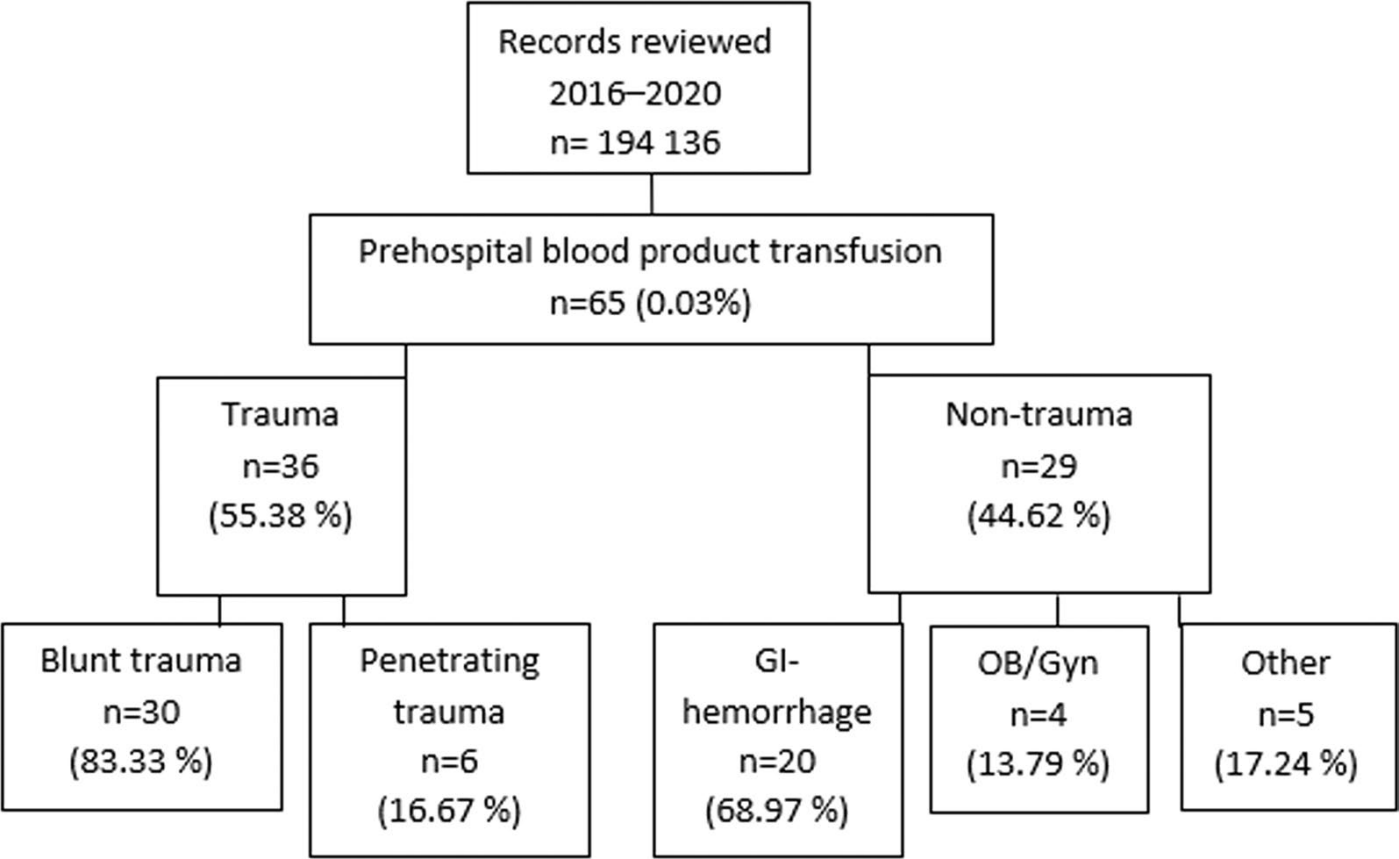
Flow chart of the study population

65 patients were identified receiving prehospital blood products during the study period (September 2016–December 2020) (Fig. 1.). Most of the transfused patients (n = 40, 62%) were men, the median age being 54 (IQR 32) years. Three (4.62%) pediatric patients (< 13 years) were transfused during the study period. The two main reasons for PHBTs were blunt trauma (n = 30, 46%) and gastrointestinal hemorrhage (n = 20, 31%).

Out of the 65 transfused patients 55% (n = 36) were trauma patients (Fig. 1). The trauma group consisted of blunt trauma (n = 30, 83%) mainly traffic accidents (n = 22, 73%), falls for height over 6 m (n = 3, 10%), falls or other impacts (n = 5, 17%) and penetrating trauma (n = 6, 17%) included five (83%) stabbings and one (17%) shooting. 78% of the trauma patients (n = 28) were men, the median age being 47 years vs. 66 years (p < 0.001) in non-traumatic patients.

The non-traumatic group (n = 29, 45%) consisted of gastrointestinal hemorrhage (GI-hemorrhage) (n = 20, 69%), obstetric/ gynecological (Ob/Gyn) (n = 4, 14%) related problems, pregnancy-related hemorrhage and other hemorrhage mechanisms (n = 5, 17%) like postoperative bleeding and suspected ruptured aortic aneurysm. More women (n = 17, 59%) were transfused for nontraumatic reasons than men (n = 12, 41%).

**Fig. 2:**
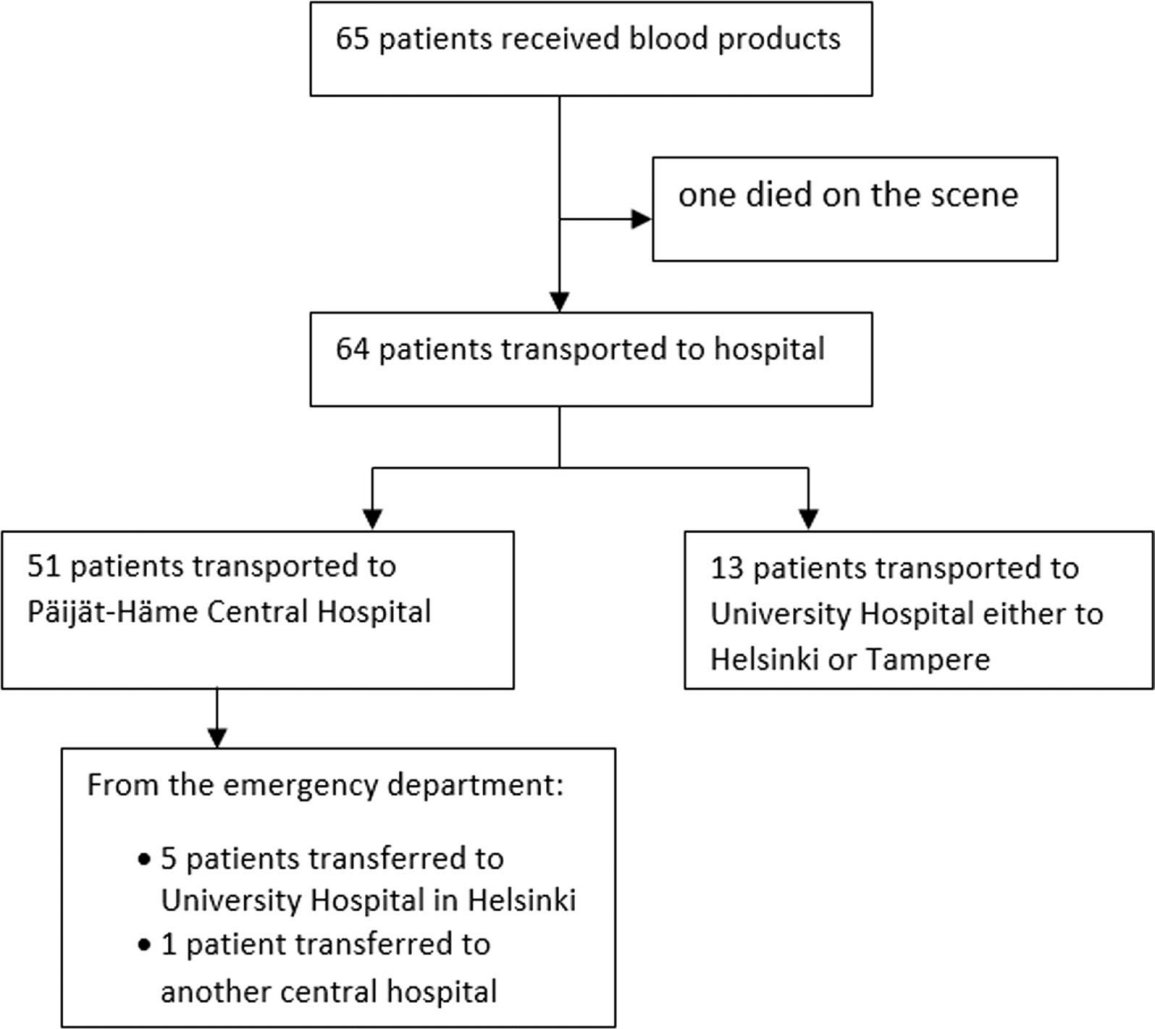
Patient transport from the scene and immediate hospital transfers

From the 65 patients treated with PHBT, one died (1.5%) on the scene, and 51 (78%) were transported to Päijät-Häme Central Hospital, and 13 (20%) were transported to the university hospital either to Helsinki or Tampere for more specialized care (Fig. 2.). Out of the 13 patients transported to the university hospital four (31%) of them were transported by HEMS unit and nine (69%) were transported by ambulances. After initial stabilization and assessment at the emergency department in Päijät-Häme Central hospital six (11.7%) patients were further transferred to other hospitals, five (9.8%) to the university hospital in Helsinki and one (1.9%) to another central hospital closer to their hometown.

### Prehospital blood product transfusions

The median time from the emergency call to the first PHBT was 54 min (IQR 38). The median time was 39 min (IQR 29) to start the transfusion after the first ambulance crew met the patient. The transfusion began on median 61 min (IQR 42) before arrival at the emergency department (Table 1). From the beginning of the transfusion five patients arrived at the emergency department under 20 min and from the start of the emergency call only 3 patients arrived at the emergency department under 60 min.

**Table 1:** Characteristics of the patients. Data presented as value (%) or as median (inter quartile range)

	Total	Trauma	Nontraumatic
Gender, male, n (%)	40 (62%)	28 (78%)	12 (41%)
Age, years (IQR)	54 (32)	47 (32)	66 (26)
Time, min (IQR)			
From the emergency call to transfusion	54 (38)	51 (33)	54 (44)
From the start of first blood product arriving to the hospital	61 (42)	64.5 (64.25)	60 (38.5)
Haemodynamic parameters (IQR)			
Lowest systolic blood pressure pretransfusion (mmHg)	76.5 (36.5)	80 (41)	70 (28)
Pretransfusion heart rate (bpm)	100 (35)	100 (30)	100 (37)
Prehospital systolic blood pressure after transfusion (mmHg)	111.5 (34.5)	117 (35.25)	105 (21.5)
Prehospital after transfusion heart rate (bpm)	95 (31)	95 (34)	94 (24.25)
Systolic blood pressure at arrival to the hospital (mmHg)	116.5 (26.5)	110 (40)	120 (19.5)
Heart rate at arrival to the hospital (bpm)	100 (22.5)	103 (11)	95.50 (27.25)
Laboratory results, hospital arrival (IQR)			
Hb (g/l)	100 (36)	125 (35)	96 (37)
pH	7.381 (0.158)	7.365 (0.132)	7.382 (0.182)
Base excess mEq/l	− 4.9 (8.8)	− 3.7 (5)	− 5.0 (9.85)
Lactate mmol/l	2.75 (4.75)	2.7 (3.9)	2.9 (7.42)
Ca-ion mmol/l	1.15 (0.0975)	1.15 (0.07)	1.14 (0.175)

During the prehospital phase, 8 (12%) patients were transfused with only RBC (3 vs. 5 in trauma/non-traumatic), 25 (38%) patients were transfused with FDP (16 vs. 9) and 32 (49%) (17 vs 15) were transfused both pRBC and FDP. TXA was administered with blood product for 94% (n = 61) of the patients. 100% (n = 29) of the non-traumatic patients got TXA with blood products vs. 89% (n = 32) of traumatic patients. Calcium gluconate was administered for 8% (n = 5) of the patients with blood products. Forty-two patients were given one unit of freeze-dried plasma and 14 patients received two units of freeze-dried plasma. Twenty-six patients were given one unit of PRBC and 14 were given two units.

### Hemodynamic variables

Before prehospital transfusion, the median systolic blood pressure was 76.5 mmHg (IQR 36.5) and after transfusion the prehospital median systolic blood pressure was 111.5 mmHg (IQR 34.5) (p < 0.001). The median systolic blood pressure at the emergency department after the prehospital transfusion was 116.6 mmHg (IQR 26.5) (p < 0.001). For trauma patients, the median systolic blood pressure before transfusion was 80 mmHg (IQR 41) and at the arrival to the hospital 110 mmHg (IQR 40) (p = 0.003). For the non-traumatic patients, the median blood pressure before transfusion was 70 mmHg (IQR 28), at arrival to the hospital the median systolic blood pressure was 120 mmHg (IQR 19.5) (p < 0.001). The patient characteristics are presented in the Table 1.

### Safety

The patients’ median temperature before transfusion was 36.1 °C (IQR 1.6 °C). At arrival to the hospital. the median temperature was 36.3 °C (IQR 1.3 °C). For 6 patients, temperature rose 1 degree or more during prehospital phase and for 1 patient temperature increased by more than 2 degrees. No transfusion-related severe adverse such as transfusion-related acute lung injury, transfusion-associated circulatory overload, allergic reactions, nor acute hemolysis, were noted during prehospital phase or reported in the hospital records.

### In-hospital blood transfusions and outcomes for patients receiving prehospital transfusion

Out of the 51 patients transported to the Päijät-Häme central hospital, one (1.96%) died on arrival and 6 patients were transferred from the emergency department to other hospitals. Before and or during the interhospital transfer, 3 (50%) patients received blood products either pRBCs or pRBCs and plasma. Out of the 44 patients treated in the Päijät-Häme central hospital, 52.3% (n = 23) received blood products during the first 24 h of care (Table 2).

**Table 2:** 6-h and 24-h blood component usage in the hospital, n = 44

Units of blood products	6-h n (%)	24-h n (%)
pRBC 1–2 units	14 (31.82%)	7 (15.91%)
pRBC 3–5 units	4 (9.09%)	12 (27.27%)
pRBC 6–9 units	2 (4.55%)	3 (6.82%)
pRBC 10 or more units	1 (2.27%)	1 (2.27%)
Platelets 1–2 units	2 (4.55%)	2 (4.55%)
Platelets 3–4 units Platelets 5–6 units	2 (4.55%) 0 (0%)	2 (4.55%) 1 (2.27%)
Plasma 1–2 units	6 (13.64%)	7 (15.91%)
Plasma 3–5 units	2 (4.55%)	3 (6.82%)
Plasma 6–9 units	2 (4.55%)	2 (4.55%)

Upon admission, 31.8% (n = 14) of the patients had an endoscopic procedure and 34.1% (n = 15) had a surgical operation during the hospital stay. Eleven patients (22%) were admitted to the intensive care unit (ICU), and 22 patients (44%) were admitted to high dependency unit (HDU) after emergency department. Eight (73%) of the eleven patients admitted to ICU were trauma patients and 6 (27%) out of the 22 patients admitted to the HDU were trauma patients. The median length of stay in the ICU was 3 days (IQR 4) and the median length of stay in the HDU was 3 days (IQR 3). The median length of stay in the hospital was 5 days (IQR 6). Out of the 45 patients treated in Päijät-Häme Central Hospital 8 (17.8%) passed away during hospital stay. Median time of death in hospital was 6.5 h (IQR 46.9). Out of the 65 patients, one (1.5%) died during prehospital phase. 37 (82.2%) patients were discharged alive either to home or to another health care facility.

### Waste of blood products

14 pRBCs were marked as wastage (2.7%). The main reason was equipment malfunction. In the beginning, pRBCs were kept in a fridge by the rapid response unit and the temperature was too cold (4 units). Since carrying pRBCs on board continuously, 4 units went to waste because the temperature sensor (Libero) in use was not on, and after switching to the Seemoto-sensor no equipment malfunctions have been reported. For 3 of the units, there was no explanation for wastage and for other 3 units that were meant to be transfused, the transfusion did not happen, but the red blood cells were already warmed up and thus waste.

## Discussion

Päijät-Häme region EMS implemented a prehospital blood transfusion protocol in the fall of 2016 and was the first civilian organization in Finland to include freeze dried plasma in the protocol. The primary aim of this study was to describe the patients that received PHBT in ground-based EMS with long transport times and secondary aims were to analyze PHBTs’ effect for patients’ physiology and further transfusion need. The main findings of our study were the following: First, the most common reasons why patients received PHBT were traffic accidents or patients suffering from gastrointestinal hemorrhage. These two patient groups were equally large and represent 65% of the patients treated with PHBT. Second, transfusion began significantly earlier than it would have started on arrival to the hospital. Third, hemodynamic parameters show improvement in both traumatic and nontraumatic patients after PHBT.

Recognizing hemorrhaging patients in need of PHBT can be challenging in a prehospital setting [13] and administering a prehospital blood transfusion is not an everyday procedure for paramedics. During the study period, 65 patients received PHBT, which equals to 1.25 patients per month. This is just 0.03% of all the region’s emergency calls. During the first 12 months five patients received PHBT compared to the last 12 months when 29 patients received PHBT. This increase (480%) can partly represent learning and implementation prosses of the new treatment protocols in EMS.

Nearly half of the patients receiving PHBT were non-traumatic, with the most common reason being gastrointestinal hemorrhage. Previous studies have reported the non-traumatic group being anywhere from 22 to 68% of the patients receiving PHBT and gastrointestinal hemorrhage being the most common reason [10, 12, 14–16]. The patient characteristics observed in our study closely echo previous research. Overall, the non-traumatic group consisted of older patients and in deeper anemia compared to the traumatic group [10, 12, 15]. The traumatic group consisted of younger, mostly males suffering blunt traumas [2, 12, 17].

The delay of starting transfusions in hemorrhaging patients at the hospital is significant compared to when transfusions are administered in the prehospital setting [2–4]. In our study, the median delay would have been around 115 min from start of the emergency call to arrival to the hospital. Similar delays have been reported also by Lyon et al. [2] 114 min delay, Vuorinen et al. [14] 103 min delay and Ångerman et al. [12] 97 min delay for non-traumatic patients and 83 min for traumatic. In this study, the PHBT was started median 54 min (IQR 38) from the emergency call and for trauma patients the median time was 51 min and for non-traumatic 54 min. These times compare well with Finnish HEMS units, for Tampere HEMS (FH30) it takes on median 71 min (IQR 34) form the start of the emergency call to start of the prehospital blood transfusion [14] and for Vantaa HEMS unit (FH10) it takes median 49 min (IQR 28) for trauma patients and 64 min (IQR 30) for non-traumatic patients [12].

Prehospital blood transfusion can be beneficial to the patients that have deep hemorrhagic shock or ongoing massive bleeding [13] and when the transport time to the hospital is longer than 20 min [17]. In shorter transport times patients do not seem to have any survival benefit [3]. Oekeshotta et al. [4] reported that transferring patients to hospital before transfusion would cost a further 71 min of delay and Vuorinen et al. [14] reported transfusions beginning 33 min before arrival to the hospital. Of the 65 patients transfused during the study period, only five patients arrived at the emergency department under 20 min from the beginning of transfusion, the median time being 61 min. For those patients, the transfusion started significantly earlier than it would have started on arrival to the hospital. The current research is not clear whether prehospital blood product transfusions benefit the patient when compared to saline. [3, 18] To recognize the patient groups that would benefit earlier blood product or whole blood transfusions needs more research.

According to previous studies, prehospital blood transfusions seem to be safe, and paramedic led teams can perform safe transfusions as well as physician led teams [19]. Serious adverse events have not been reported [3, 7, 8, 10, 20–22]. Mild transfusion reactions vary in between 0.1% [10] and 2.2% [20]. Some of the reported reactions have been connected to fresh frozen plasma transfused in the hospital [10, 21]. PHBT can decrease trauma patients need for blood products during the first 24 h of care [5, 7–9] and this can potentially decrease patients allogeneic tissue exposure and optimize the use of limited resource [7]. In our study, no transfusion-related severe adverse events were noted during or after prehospital phase. A mild fever reaction is defined as a patients’ temperature increase of over 1 °C from the pretransfusion temperature or over 38 °C during the transfusion or a 4-h period after transfusion. A severe fever reaction is an immediate temperature increase of more than 2 °C [23]. Six patients’ temperature increased 1 °C or more during the prehospital phase and 1 patient had a temperature increase of more than 2 °C but was hypothermic before transfusion and normothermic on arrival to the hospital. Preventing and treating hypothermia is part of the trauma protocol during the prehospital phase. Since hypothermia increases mortality in trauma patients [24–27], the active use of thermal protection and warm fluids benefits patients [25, 26]. Recognizing transfusion-related adverse events can be complicated in a prehospital setting, where patients’ clinical status can simulate transfusion reactions [16] and treatments can further confound the findings.

## Limitations

The limitations of this study were as follows: First, this study presents a small number of PHBT patients from one ground-based EMS system, in one hospital district area, and as such the findings may not apply to other prehospital systems or hospital districts. As a single-center study design, the number of patients was small and thus limits the statistical significance, however, we can be certain that we included all transfused patients. Second, the data was collected retrospectively from both prehospital and hospital records and may therefore contain unintentional bias. Some of the data was incomplete in both prehospital and hospital records, and for the patients transported straight to the university hospital we do not have any data from the hospital phase of care. Third, this study presents only the patients that received PHBT without comparison group to determine differences between traditional treatment and new PHBT protocol. Fourth, patients’ coagulation status on arrival to the hospital was not collected from the hospital records nor was it determent before transfusion in the prehospital setting. Fifth, data regarding crystalloid administration was not used. Currently it is difficult to determine the exact quantity of crystalloids infused from the prehospital records. This would be an estimation to the closest 500–1000 ml and as such would not always be realistic representation of the true amount patients received. In the future, larger multi-center, and prospectively designed studies could bring more information to the clinical decision-making behind the PHBT and of the patient’s coagulation status in the prehospital stage and on arrival to the hospital.

## Conclusions

This study finds out that starting PHBT in a ground-based emergency medical service is a feasible option and can decrease the time to transfusion significantly. The majority of transfused patients were either suffering from gastrointestinal hemorrhage or patients from traffic accidents. Although these two groups had differences in their hemodynamic parameters and laboratory results, all the patients’ showed improvement in their vital signs after PHBT. There were no transfusion-related serious adverse events noted in the records, therefore making the PHBT in a ground-based emergency medical service appear to be safe. More research is needed on patient recognition, universal triggering values and decision-making rules for PHBT and transfusion safety protocols in prehospital setting and paramedics role on prehospital blood transfusions.

## Data Availability

The datasets used and/or analyzed during the current study are available from the Päijät-Häme Joint Authority for Health and Wellbeing on reasonable request compliance with the General data protection regulation and Finnish legislation.

## Abbreviations

pRBCs: Packed red blood cells
EMS: Emergency medical service
PHBT: Prehospital blood product transfusion
FDP: Freeze-dried plasma
PHEC: Prehospital emergency care
ICU: Intensive care unit
HDU: High dependency unit
HEMS: Helicopter emergency medical service
TXA: Tranexamic acid
IQR: Interquartile range
OB/Gyn: Obstetric/gynecological
GI-hemorrhage: Gastrointestinal hemorrhage

## References

[1] Harris T, Davenport R, Mak M, Brohi K (2018). The evolving science of trauma resuscitation. Emerg Med Clin North Am.

[2] Lyon RM, de Sausmarez E, McWhirter E, Wareham G, Nelson M, Matthies A (2017). Pre-hospital transfusion of packed red blood cells in 147 patients from a UK helicopter emergency medical service. Scand J Trauma Resusc Emerg Med.

[3] Moore HB, Moore EE, Chapman MP, McVaney K, Brykiewicz G, Blechar R (2018). Plasma-first resuscitation to treat haemorrhagic shock during emergency ground transportation in an urban area: a randomised trial. Lancet.

[4] Oakeshotta JE, Griggsa JE, Warehama GM, Lyona RM, on behalf of Kent Surrey Sussex Air Ambulance Trust (2019). Feasibility of prehospital freeze-dried plasma administration in a UK helicopter emergency medical service. Eur J Emerg Med.

[5] Holcomb JB, Donathan DP, Cotton BA, del Junco D, Brown G, von Wenckstern T (2015). Prehospital transfusion of plasma and red blood cells in trauma patients. Prehosp Emerg Care.

[6] Guyette FX, Sperry JL, Peitzman AB, Billiar TR, Daley BJ, Miller RS (2021). Prehospital blood product and crystalloid resuscitation in the severely injured patient a secondary analysis of the prehospital air medical plasma trial. Ann Surg.

[7] Rehn M, Waver E, Eshelby S, Røislien J, Lockey J (2018). Pre-hospital transfusion of red blood cells in civilian trauma patients. Transfus Med.

[8] Griggs JE, Jeyenathan J, Joys M, Russell MQ, Durgei N, Bootlands D (2018). Mortality of civilian patients with suspected traumatic haemorrhage receiving prehospital transfusion of packed red blood cells compared to pre-hospital crystalloid. Scand J Trauma Resusc Emerg Med.

[9] van Turenhout EC, Bossers SM, Loer SA, Giannakopeulos GF, Schwarter LA, Schober P (2020). Pre-hospital transfusion of red blood cells. Part 2: a systematic review of treatment effects on outcomes. Transfus Med.

[10] Thiels CA, Aho JM, Fahy AS, Parker ME, Glasgow AE, Berns KS (2016). Prehospital blood transfusions in non-trauma patients. World J Surg.

[11] Parker ME, Khasawneh MA, Thiels CA, Berns KS, Stubbs JR, Jenkins DH (2017). Prehospital transfusion for gastrointestinal bleeding. Air Med J.

[12] Ångerman S, Kirves H, Nurmi J (2021). Characteristics of nontrauma patients receiving prehospital blood transfusion with the same triggers as trauma patients: a retrospective observational cohort study. Prehosp Emerg Care.

[13] van Turenhout EC, Bossers SM, Loer SA, Giannakopoulos GF, Schwarte LA, Schober P (2020). Pre-hospital transfusion of red blood cells. Part 1: a scoping review of current practice and transfusion triggers. Transfus Med.

[14] Vuorinen P, Kiili J-E, Setälä P, Kämäräinen A, Hoppu S (2020). Prehospital administration of blood products: experiences from a Finnish physician-staffed helicopter emergency medical service. BMC Emerg Med.

[15] Cassingnol A, Marmin J, Mattei P, Goffinet L, Pons S, Renard A (2020). Civilian prehospital transfusion—experiences from a French region. Vox Sang.

[16] Sunde GA, Vikenes B, Strandenes G, Flo K-C, Hervig TA, Kristoffersen EK (2015). Freeze dried plasma and fresh red blood cells for civilian prehospital hemorrhagic shock resuscitation. J Trauma Acute Care Surg.

[17] Pusateri AE, Moore EE, Moore HB, Le TD, Guyette FX, Chapman MP (2020). Association of prehospital plasma transfusion with survival in trauma patients with hemorrhagic shock when transport times are longer than 20 minutes a post hoc analysis of the PAMPer and COMBAT clinical trials. JAMA Surg.

[18] Crombie N, Doughty HA, Bishop JRB, Desai A, Dixon EF, Hancox JM (2022). Resuscitation with blood products in patients with trauma-related haemorrhagic shock receiving prehospital care (RePHILL): a multicentre, open-label, randomised, controlled, phase 3 trial. Lancet Haematol.

[19] Shand S, Curtis K, Dinh M, Burns B (2019). What is the impact of prehospital blood product administration for patients with catastrophic haemorrhage: an integrative review. Int J Care Inj.

[20] Sperry JL, Guyette FX, Brown JB, Yazer MH, Triulzi DJ, Early-Young BJ (2018). Prehospital plasma during air medical transport in trauma patients at risk for hemorrhagic shock. N Engl J Med.

[21] Peters JH, Smulders PSH, Moors XRJ, Bouman SJM, Meijis CMEM, Hoogerwerf N (2019). Are on-scene blood transfusions by a helicopter emergency medical service useful and safe? A multicentre case–control study. Eur J Emerg Med.

[22] Rijnhout TWH, Wever KE, Marinus RHAR, Hoogerwerf N, Geeraedts LMG, Tan ECTH (2019). Is prehospital blood transfusion effective and safe in haemorrhagic trauma patients? A systematic review and meta-analysis. Injury.

[23] Finnish Red Cross Blood Services. Blood Transfusion Reactions. https://www.bloodservice.fi/for-health-care-professionals/blood-transfusions/blood-transfusion-reactions (2021). Accessed 6.9.2021

[24] Aitken LM, Hendrikz JK, Dulhunty JM, Rudd MJ (2009). Hypothermia and associated outcomes in seriously injured trauma patients in a predominantly sub-tropical climate. Resuscitation.

[25] Perlman R, Callum J, Laflamme C, Tien H, Nascimento B, Beckett A (2016). A recommended early goal-directed management guideline for the prevention of hypothermia-related transfusion, morbidity, and mortality in severely injured trauma patients. Crit Care.

[26] Lapostolle F, Couvreur J, Koch FX, Savary D, Alhéritière A, Galinski M (2017). Hypothermia in trauma victims at first arrival of ambulance personnel: an observational study with assessment of risk factors. Scand J Trauma Resusc Emerg Med.

[27] Rösli D, Schnüriger B, Candinas D, Hatmeier T (2020). The impact of accidental hypotermia on mortality in trauma patients overall and patients with traumatic brain injury specifically: a systematic review and meta-analysis. World J Surg.

